# Comparative Analysis of Circulating Endothelial Progenitor Cells in Age-Related Macular Degeneration Patients Using Automated Rare Cell Analysis (ARCA) and Fluorescence Activated Cell Sorting (FACS)

**DOI:** 10.1371/journal.pone.0055079

**Published:** 2013-01-24

**Authors:** Emil Anthony T. Say, Alex Melamud, Denise Ann Esserman, Thomas J. Povsic, Sai H. Chavala

**Affiliations:** 1 Kittner Eye Center, University of North Carolina Hospitals, Chapel Hill, North Carolina, United States of America; 2 Retina Group of Washington, Washington, D.C., United States of America; 3 Departments of Medicine, Division of General Medicine and Clinical Epidemiology and Biostatistics, Gillings School of Global Public Health, University of North Carolina, Chapel Hill, North Carolina, United States of America; 4 Duke Clinical Research Institute and Department of Medicine, Duke University Medical Center, Durham, North Carolina, United States of America; Massachusetts Eye & Ear Infirmary, Harvard Medical School, United States of America

## Abstract

**Background:**

Patients with age-related macular degeneration (ARMD) begin with non-neovascular (NNV) phenotypes usually associated with good vision. Approximately 20% of NNV-ARMD patients will convert to vision debilitating neovascular (NV) ARMD, but precise timing of this event is unknown. Developing a clinical test predicting impending conversion to NV-ARMD is necessary to prevent vision loss. Endothelial progenitor cells (EPCs), defined as CD34^+^VEGR2^+^ using traditional fluorescence activated cell sorting (FACS), are rare cell populations known to be elevated in patients with NV-ARMD compared to NNV-ARMD. FACS has high inter-observer variability and subjectivity when measuring rare cell populations precluding development into a diagnostic test. We hypothesized that automated rare cell analysis (ARCA), a validated and FDA-approved technology for reproducible rare cell identification, can enumerate EPCs in ARMD patients more reliably. This pilot study serves as the first step in developing methods for reproducibly predicting ARMD phenotype conversion.

**Methods:**

We obtained peripheral venous blood samples in 23 subjects with NNV-ARMD or treatment naïve NV-ARMD. Strict criteria were used to exclude subjects with known angiogenic diseases to minimize confounding results. Blood samples were analyzed in masked fashion in two separate laboratories. EPCs were independently enumerated using ARCA and FACS within 24 hours of blood sample collection, and p<0.2 was considered indicative of a trend for this proof of concept study, while statistical significance was established at 0.05.

**Results:**

We measured levels of CD34^+^VEGFR2^+^ EPCs suggestive of a trend with higher values in patients with NV compared to NNV-ARMD (p = 0.17) using ARCA. Interestingly, CD34^+^VEGR2^+^ EPC analysis using FACS did not produce similar results (p = 0.94).

**Conclusions:**

CD34^+^VEGR2^+^ may have predictive value for EPC enumeration in future ARCA studies. EPC measurements in a small sample size were suggestive of a trend in ARMD using ARCA but not FACS. ARCA could be a helpful tool for developing a predictive test for ARMD phenotype conversion.

## Introduction

**Figure 1:**
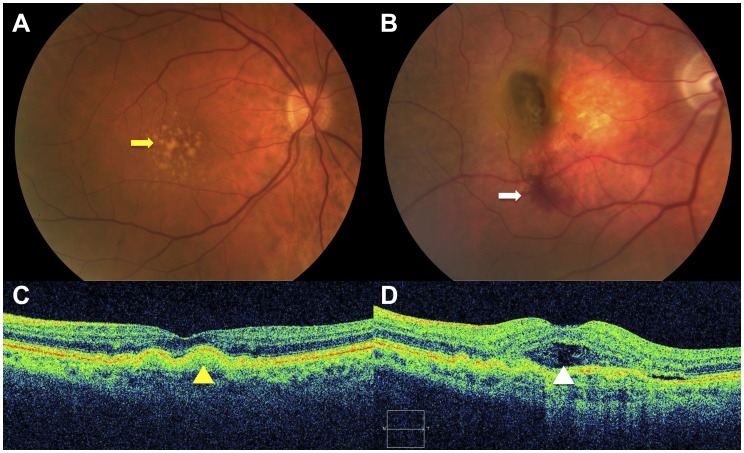
Representative images of age-related macular degeneration (ARMD). ARMD is a clinical spectrum that begins with accumulation of yellowish deposits (yellow arrow) underneath the retinal pigment epithelium (RPE) called drusen (A), which are hallmarks of non-neovascular ARMD. In some patients, this may progress to advanced disease with subretinal hemorrhage (white arrow) from choroidal neovascularization in neovascular ARMD (B). Optical coherence tomography (C–D) is an imaging modality frequently used to distinguish the two entities. Smooth dome-shaped elevations of the RPE (yellow arrowhead) typically correspond to drusen seen on clinical examination in non-neovascular ARMD (C). When subretinal fluid accumulates beneath the neurosensory retina (white arrowhead), this is indicative of conversion to neovascular ARMD (D).

Age-related macular degeneration (ARMD) is the leading cause of vision loss in the Western world. Its clinical spectrum is diverse, beginning with yellowish deposits beneath the retina called drusen in non-neovascular age-related macular degeneration (NNV-ARMD) and advancing to choroidal neovascularization, subretinal hemorrhage, and scarring in neovascular age-related macular degeneration (NV-ARMD). (Figure 1A–B) In clinical practice, management of ARMD begins with differentiating between NNV-ARMD and NV-ARMD using clinical examination and imaging tests. Intravenous fluorescein angiography and optical coherence tomography are imaging modalities that allow identification of choroidal neovascular membranes and accumulation of subretinal fluid in NV-ARMD. (Figure 1C–E).

Circulating endothelial progenitor cells (EPC) contribute to pathologic angiogenesis in NV-ARMD in a process that recapitulates developmental vasculogenesis. [1]–[3] Rodent models of choroidal neovascularization and human autopsy samples confirm the presence of EPCs in subretinal neovascular complexes. EPCs were also found to be elevated in subjects with both NNV-ARMD and NV-ARMD using various methods. [4]–[6] Thill et. al. used peripheral blood mononuclear cell cultures to detect late outgrowth EPCs and found them to be significantly elevated in patients with high-risk NNV-ARMD and even higher in those with NV-ARMD. [5] A similar study by Machalinska et. al. using flow cytometry found elevated EPCs in ARMD patients compared to healthy adults. [6].

**Figure 2:**
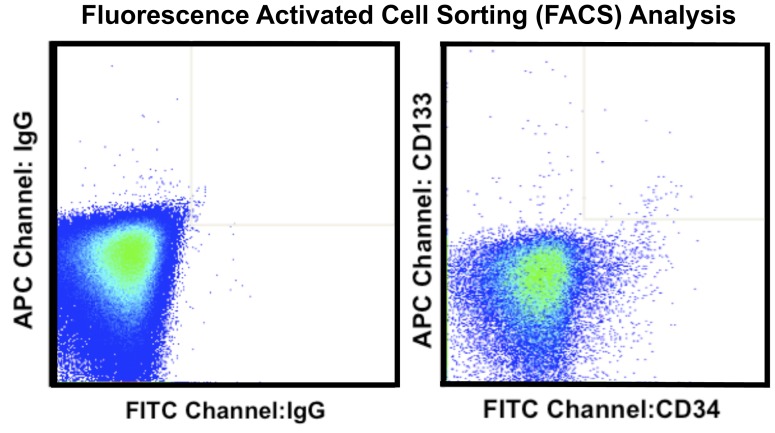
Representative fluorescence activated cell sorting (FACS) analysis.

**Figure 3:**
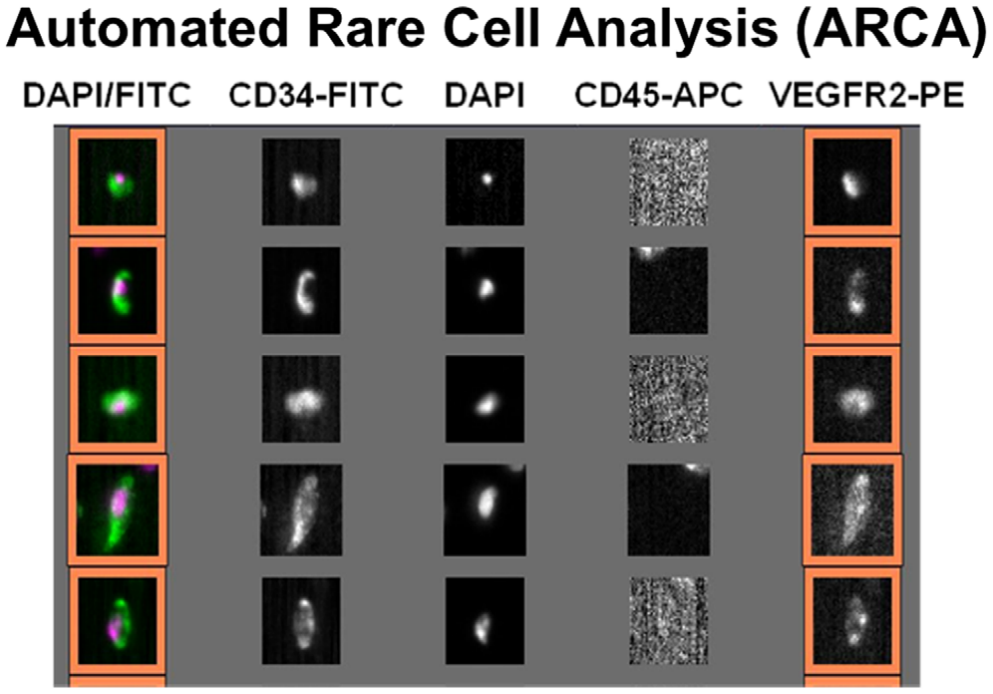
Representative CellTracks Analyzer II analysis.

Although several studies demonstrate a strong correlation between EPCs and ARMD phenotype, the clinical applications are limited due to laborious detection procedures, subjective operator-dependent techniques, and lack of reproducibility. [7] The high inter- and intra-observer variability associated with traditional techniques such as fluorescence activated cell sorting (FACS) analysis are attributed mainly to differences in sample preparation and analysis techniques. [8] (Figure 2) Automated rare cell analysis (ARCA) attempts to overcome these limitations. One example is the CellSearch System (Veridex, Raritan, NJ), which uses standard collection tubes (CellSave, Veridex, Raritan, NJ) for preserving and transporting blood samples providing an opportunity for wide-spread implementation and remote analysis. The CellSearch System uses automated immunomagnetic and immunofluorescent labeling to identify and enumerate specific rare cell populations in peripheral blood. This minimizes operator dependency, and enhances reproducibility, and providing ease of use and immediate results for translation to clinical practice. [9] (Figure 3) This technology is FDA approved for predicting progression free survival in metastatic breast, colorectal and prostate cancer, and has been validated for detection of circulating EPCs in sickle cell disease and acute myocardial infarction. [9]–[12].

As a proof of concept, we hypothesized that ARCA can be used to identify and enumerate EPCs, defined as CD34^+^VEGFR2^+^CD45^–^ peripheral blood mononuclear cells, in patients with ARMD similar to previous studies using FACS and peripheral blood mononuclear cell cultures. We also analyzed the samples for other cell surface markers of EPCs and hematopoietic stem cells.

## Materials and Methods

Subjects 50 years and older with a clinical diagnosis of unilateral NV-ARMD or bilateral Age-related Eye Disease Study (AREDS) categories 1–3 NNV-ARMD without geographic atrophy seen at the Duke University Eye Center and Retina Group of Washington from July 2009 to March 2011 were eligible for this study. [13] The methods adhered to the Declaration of Helsinki and the study was approved by the Institutional Review Board of Duke University, University of North Carolina, and Retina Group of Washington. All subjects demonstrated understanding of the research study and signed written informed consents before clinical examination and blood extraction. Only treatment naïve NV-ARMD subjects were included. Subjects with medical conditions that may effect EPC levels apart from ARMD such as stroke, cardiovascular disease, diabetes mellitus, liver disease, premenopausal state, and cancer, or ocular diseases that may produce retinal or subretinal neovascularization like diabetic retinopathy and arterial or vein occlusion, were excluded. All patients underwent a complete medical history review; in addition, smoking status, as well as statin, ACE inhibitor, anti-coagulant, and AREDS supplement use were recorded.

Subjects had a comprehensive ophthalmologic examination, including best-corrected visual acuity, slit lamp examination and biomicroscopy, and optical coherence tomography. Intravenous fluorescein angiography (IVFA) was performed at the discretion of the examiner if choroidal neovascularization was suspected.

A follow-up questionnaire was administered at least 6 months after the initial visit to determine if there was a new vascular event, conversion to NV-ARMD, or a newly diagnosed ocular disease.

### Sample Collection

Two separate 4 ml samples, one ethylenediaminetetraacetic acid (EDTA) blood and one CellSave (Veridex, Raritan, NJ) preserved blood, were obtained by venous puncture after 3 ml of blood was wasted to avoid collection of endothelial cells from venipuncture. In some cases, IVFA was performed prior to blood collection. (See Table 1) For EPC enumeration, samples were sent to a single laboratory at Duke University (Durham, NC) for traditional FACS, while CellSave preserved samples were sent to and analyzed by Veridex, LLC (Huntingdon Valley, PA) using CellTracks AutoPrep System and CellTracks Analyzer II (Veridex, Raritan, NJ). All samples were sent overnight and analyzed independently within 24 hours of blood extraction.

**Table 1:** Patient characteristics.

Characteristics	Non-neovascular ARMD, n = 15 (%)	Neovascular ARMD, n = 8 (%)	p-value
Median age (years), range	72, 52–86	78, 67–86	0.1453
Sex			0.1930
Male	9 (60)	2 (25)	
Female	6 (40)	6 (75)	
Smoking history^1^	4 (27)	2 (25)	1
Medications			
Statins	5 (33)	4 (50)	0.6570
ACE inhibitors	1 (7)	1 (13)	1
Anticoagulants	5 (33)	5 (63)	0.2213
AREDS 1 supplement	10 (67)	1 (13)	0.0272
IVFA before blood draw	4 (27)	8 (100)	0.0013
Median follow-up (months), range^2^	18, 6–28	16.5, 7–24	0.5224
New CNV	0 (0)	0 (0)	
New vascular event	1 (7)	0 (0)	

* ARMD – age-related macular degeneration, CNV – choroidal neovascularization, IVFA – intravenous fluorescein angiography, AREDS 1– Age-related Eye Disease Study 1^13.^

1 Smoking history defined as patients who admitted to smoking at the time of blood extraction or has quit <10 years prior.

2 1 patient with non-neovascular ARMD and 2 patients with neovascular ARMD did not complete follow-up questionnaires.

### Non-automated Fluorescence Activated Cell Sorting (FACS) Analysis

Blood collected into EDTA tubes was subjected to centrifugation (1800 g×20 min) to separate the plasma, red blood cells, and buffy coat layers. After plasma removal, the buffy coat was transferred to a 50 ml conical tube and isolated mononuclear cells were washed with Iscove’s Modified Dulbecco’s Medium (IMDM) containing 2% fetal calf serum (IMDM+2%) concentrated in 10^7^ cells/ml in 80 µl IMDM+2%. Non-specific antibody binding was inhibited using FcR reagent (Becton Dickinson, 10 µl×10 minutes), and cells were incubated with CD133- allophycocyanin (Miltenyi Biotec, Auburn, CA), CD34-fluorescein isothiocyanate (Miltenyi Biotec), and VEGFR2-phycoerythrin (R and D Systems, Minneapolis MN) for 60 minutes at 4°C. Dead and dying cells were excluded using 7-amino-actinomycin D (7-AAD, Invitrogen, Carlsbad, CA) added just prior to FACS.

Total human peripheral blood was analyzed for the relative content of cells with low orthogonal light scatter and high ALDH activity content (side scatter low [SSC^lo^] ALDH^br^ cells).^8^ 4×10^6^ cells were aliquoted for analysis into an Aldecount tube containing 2 mL of Aldecount buffer (Aldagen Inc., Durham, NC). Immediately after addition of the cells, 500 µL was transferred to a tube containing diethylaminobenzaldehyde (DEAB), a potent inhibitor of ALDH activity (10 µM). After 30 minutes at 37°C, the cells were centrifuged, placed on ice, and FACS was performed.

FACS was performed by trained technicians masked to subject identity using an LSR CANTO flow cytometer (BD Biosciences, San Jose, CA) and analyzed using Flow Jo software (Treestar, Costa Mesa, CA). Quality control measures were performed daily using BD Comp Beads (BD Biosciences) incubated with each antibody.

Analysis was performed in a masked fashion. Dead and dying cells were excluded based on staining with 7-aminoactinomycin D. The numbers of CD34^+^, CD133^+^, and VEGFR-2^+^ cells were identified as subpopulations of a mononuclear cell gate, determined on the basis of light front and side scatter characteristics. The number of cells staining for combinations of these markers was determined by gating on individual markers in sequential fashion. Reported frequencies were expressed as percentages of the mononuclear cell population. Determination of the ALDH^br^ populations was performed based on a comparison of staining in the FITC channel and identification of a low side-scatter high ALDH activity cluster of cells absent in a control tube for each patient containing DEAB, the ALDH inhibitor. (Figure 2).

### Automated Rare Cell Analysis of Endothelial Progenitor Cells

Enumeration of circulating EPCs was performed using the CellTracks AutoPrep and CellTracks Analyzer II System (Veridex, Raritan, NJ). Preserved blood from CellSave tubes was incubated with ferrofluids conjugated to CD34 and VEGFR2 antibodies to capture cells, while nucleic acids were stained with 4′,6-diamidino-2-phenylindole (DAPI) in the CellTracks AutoPrep system. The resulting magnetically enriched and fluorescent-labeled cells were then inserted into the MagNest Cell Preservation Device (Veridex, Raritan, NJ), which consists of a cartridge positioned between two magnets for orientation of the fluorescent and magnetically labeled cells in a monolayer to be placed into the CellTracks Analyzer II system.

The CellTracks Analyzer II is a semi-automated fluorescent microscope that captures images for each of the four fluorescent filter cubes covering the entire surface of the cartridge. Captured images containing FITC and DAPI positive events are presented in a gallery for analysis and classification of cells by the user based on cell fluorescence and morphology. (Figure 3) The cells were then identified with monoclonal antibodies against CD34 and VEGFR2 both separately and in combination. Any image positive for CD34, VEGFR2, and DAPI, as well as negative for CD45 is counted as a CD34^+^VEGFR2^+^ endothelial progenitor cells. For the study, only levels mononuclear cells immunophenotypically defined as CD34^+^, VEGFR2^+^, and CD34^+^VEGFR2^+^ were used.

### Statistical Analysis

Statistical principles were not used to estimate sample size or statistical power. Clinical data between NNV-ARMD and NV-ARMD groups were compared using the Wilcoxon rank sum test (continuous data) or Fisher’s exact test (categorical data). Differences in the number of circulating EPCs between the two groups for all cell surface markers were assessed using the Wilcoxon rank sum test. Statistical significance was established at 0.05. However, given that this was a pilot study with limited power and aimed at generating hypotheses, any p-values <0.2 were suggestive of a trend and indicative of future study.

## Results

Twenty-seven patients were recruited during the study period; four were excluded upon further review of their medical history. The study sample included 23 patients, 8 of whom had NV-ARMD. One patient with NV-ARMD had a clotted CellSave sample and was unable to undergo ARCA. There were 9 patients, 5 with NV-ARMD, who underwent intravenous fluorescein angiography prior to blood sampling, resulting in markedly higher levels of background cell fluorescence in the FITC channel. In these patients cell surface expression of CD34 and analysis of EPCs based on ALDH activity could not be performed. [14] This was not seen with ARCA. Three patients were not able to complete follow up questionnaires, 2 of which had NV-ARMD. The median ages of patients in the NNV-ARMD and NV-ARMD groups were 72 and 78 years respectively. Both groups were similar except there were more patients taking AREDS 1 supplements in the NNV-ARMD group and more who had IVFA in the NV-ARMD group. After a median follow-up of 16.5 and 18 months respectively in the NV-ARMD and NNV-ARMD groups, new choroidal neovascularization was not seen in either group; however, one patient with NNV-ARMD developed myocardial infarction a year later. (Table 1).

### Endothelial Progenitor Cell Levels

**Figure 4:**
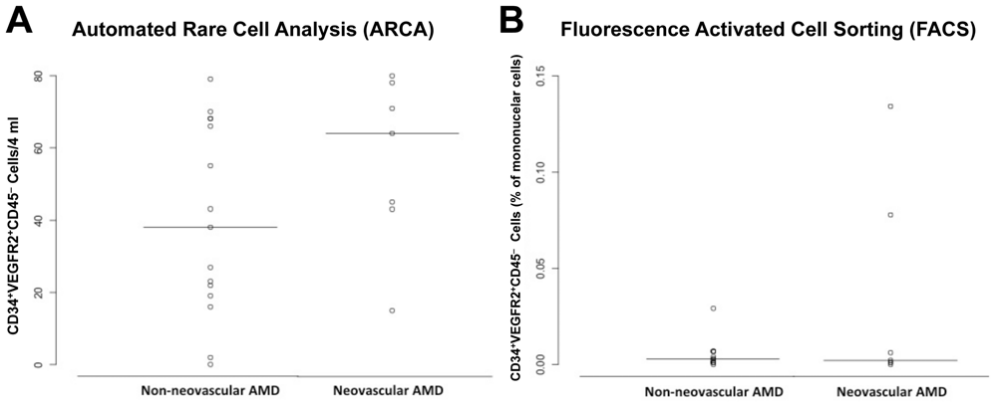
Circulating CD34^+^VEGFR2^+^CD45^–^ cells in age-related macular degeneration (ARMD). Scatter plot comparing numbers of circulating CD34^+^VEGFR2^+^ cells corresponding to endothelial progenitor cells in non-neovascular and neovascular ARMD using automated quantification (A) and traditional fluorescence activated cell sorting analysis (FACS) (B). There was a trend with more CD34^+^VEGFR2^+^CD45^–^ endothelial progenitor cells in neovascular ARMD when automated quantification was used (p = 0.17), this was not seen with FACS (p = 0.94). Horizontal solid line within scatterplots signifies the mean in each group.

**Figure 5:**
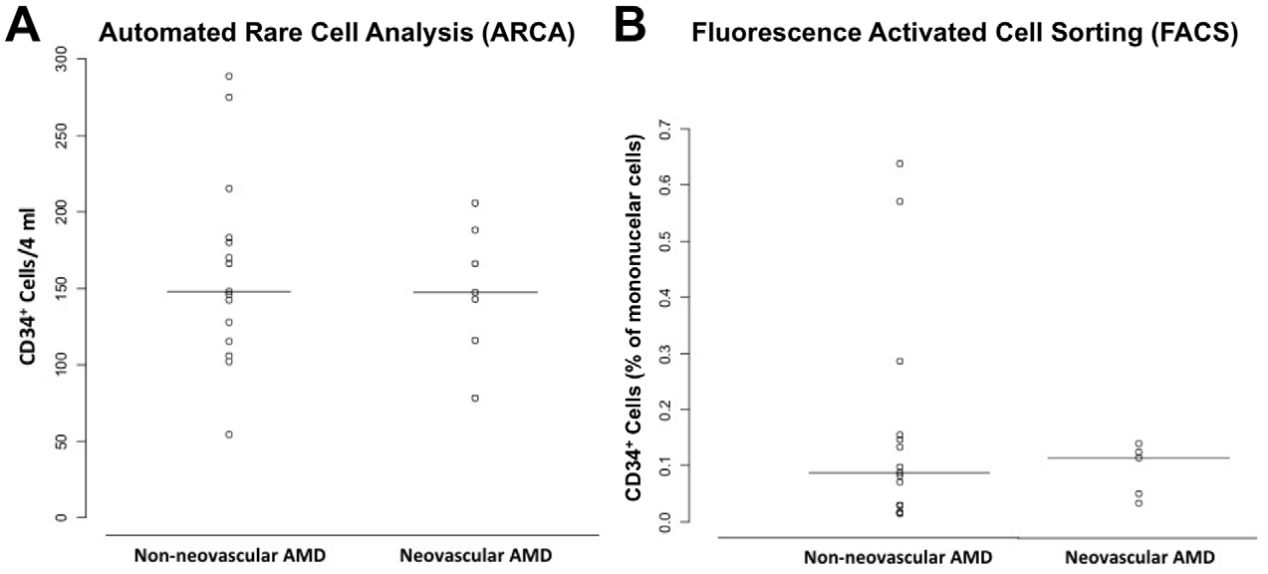
Circulating CD34^+^ cells in age-related macular degeneration (ARMD). Scatter plot comparing numbers of circulating CD34^+^ cells in non-neovascular ARMD and neovascular ARMD using automated quantification (A) and traditional fluorescence activated cell sorting analysis (FACS) (B). There was no statistical significance in the differences using either technique. Horizontal solid line within scatterplots signifies the mean in each group.

**Figure 6:**
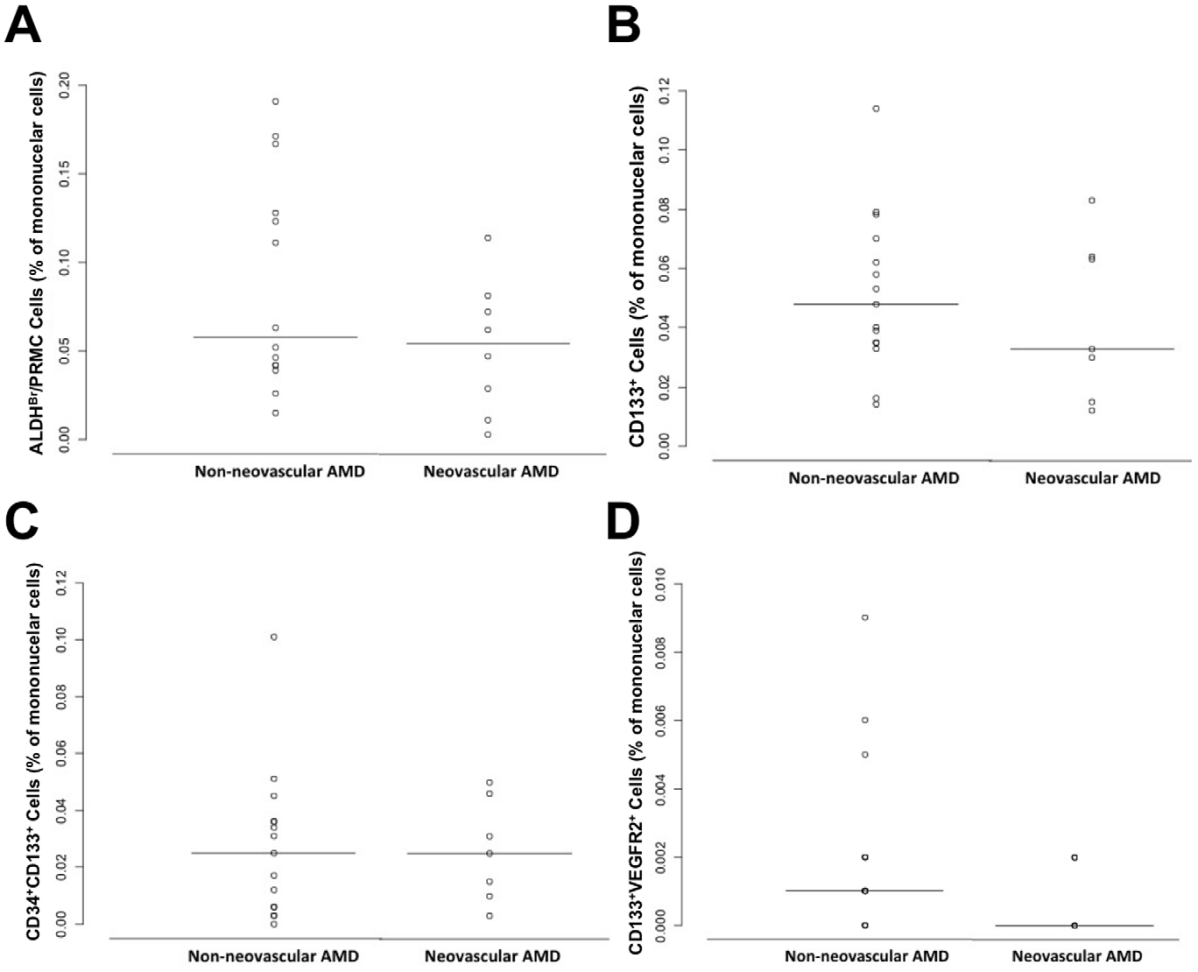
Circulating ALDH^Br^, CD133^+^, CD34^+^CD133^+^, and CD133^+^VEGFR2^+^ cells in age-related macular degeneration (ARMD). Scatter plot comparing numbers of circulating ALDH^Br^ (A), CD133^+^ (B), CD34^+^CD133^+^ (C), and CD133^+^VEGFR2^+^ (D) cells in non-neovascular and neovascular ARMD using traditional fluorescence activated cell sorting analysis (FACS). There was no statistically significant difference in surface markers analyzed using FACS between non-neovascular and neovascular ARMD. Horizontal solid line in scatterplots signifies the mean in each group.

There was an increase in the numbers of CD34^+^VEGFR2^+^CD45^–^ cells in the NV-ARMD group as assessed using ARCA (p = 0.17). This was not seen in traditional flow cytometry (p = 0.94). (Figure 4) The number of isolated CD34^+^ cells was not different in either group (ARCA, p = 0.92 or FACS, p = 1.00). (Figure 5) In addition, no difference in the two groups was seen in the numbers of ALDH^br^, CD133^+^, CD34^+^CD133^+^ and CD133^+^VEGFR2^+^ peripheral blood mononuclear cells as determined by traditional flow cytometry (p = 0.29, p = 0.46, p = 0.89, and p = 0.29 respectively). (Figure 6).

## Discussion

In this pilot study, we have demonstrated that ARCA can be used to detect differential levels of CD34^+^VEGFR-2^+^CD45^–^ EPCs in patients with NNV-ARMD and compared with NV-ARMD (p = 0.17), although future studies will be needed to confirm these results.

Until now, there is no proven therapy for NNV-ARMD progression, and management is limited to observation and reduction of modifiable risk factors. AREDS has shown that categorizing patients based on clinical findings allows stratification according to risk of progression to NV-ARMD in 5 years. Genetic testing allows determination of lifetime risk of NV-ARMD. Taken together, these criteria are helpful in determining risk, but of limited value in developing an intervention because the window for conversion is still broad. [13], [15]–[16] While some authors have combined clinical and genetic studies to create risk models to improve identification of populations at risk, it still does not address these limitations. [15]–[16] The primary goal of this study was to show that ARCA could be used in ARMD to detect and analyze EPCs. In the future, we are interested in identifying cell types and methodology that can be used to prospectively predict the conversion from NNV-ARMD to NV-ARMD. The results can then be used in conjunction with current prediction models to shorten the window for early intervention.

The present study shows high inter-subject variability, which we have noticed across all cell surface marker measurements. Some subjects may have higher circulating cells at baseline compared to others, so serial measurements will be helpful in tailoring analyses to individual subjects. This variability has been reported with traditional FACS, but appears less with ARCA. [17] Another explanation is that subjects with high EPC numbers in the high risk NNV-ARMD group may be at higher risk for developing NV-ARMD or other angiogenic events in the future. We provided a survey at six months or the next standard of care follow-up visit but it is conceivable that these subjects are at risk beyond this time period. Finally, although cell surface markers used to detect EPCs (CD34^+^VEGFR2^+^CD45^–^) were similar in both FACS and ARCA, the nuclear markers used to identify mononuclear cells in FACS (7-AAD) and ARCA (DAPI) were different. We realize it is possible this would affect the capabilities of the two methods to detect trends, but we feel that this is unlikely.

Our work has shown ARCA can detect a trend in CD34^+^VEGFR2^+^CD45^–^ EPCs between NV-ARMD and NNV-ARMD subjects, and this should serve as a foundation for moving forward with prospective trials. Our study was not powered to determine statistical significance between NV-ARMD and NNV-ARMD. Hence, we feel a prospective trial associating high-risk clinical features as defined by AREDS, genetic testing, and circulating EPCs over time can provide crucial information in identifying and possibly preventing ARMD phenotype conversion. Also, a prospective trial correlating EPCs for NV-ARMD patients undergoing anti-VEGF pharmacotherapy may serve as a surrogate biomarker for NV-ARMD activity, and guide treatment strategies and alleviate intravitreal injection burden.

Our study did not test for cell surface markers such as CD133 and ALDH^Br^ using ARCA that we performed for FACS. While this study showed an early trend analyzing CD34^+^VEGFR2^+^CD45^–^ cells using ARCA compared to FACS, the study was not designed to compare other analyzed markers in a head-to-head fashion. It is important to note that interference from fluorescein dye in patients who had blood drawn after IVFA was noted in our FACS analysis with single antibody studies, and has been reported previously with FACS. [14] To overcome this obstacle, single antibody studies were analyzed using a wavelength outside of the fluorescein dye spectrum once this was detected. A similar issue was not seen with ARCA analysis.

In summary, ARCA is immediately available for clinical use with samples available for processing within 24 hours. CD34, VEGFR2, and CD45 may be used as cell surface markers for EPC determination in futures studies using ARCA. A trend was observed measuring EPCs with ARCA but not traditional FACS.

## References

[1] Brunner S, Schernthaner GH, Satler M, Elhenicky M, Hoellerl F, et al (2009) Correlation of different circulating endothelial progenitor cells to stages of diabetic retinopathy: first in vivo data. Invest Ophthalmol Vis Sci 50: 392–398. 18719083 10.1167/iovs.08-1748

[2] Machalinska A, Modrzejewska M, Kotowski M, Dziedziejko V, Kucia M, et al (2010) Circulating stem cell populations in preterm infants. Implications for the development of retinopathy of prematurity. Arch Ophthalmol 128(10): 1311–1319. 20938001 10.1001/archophthalmol.2010.221

[3] Otani A, Kinder K, Ewalt K, Otero FJ, Schimmel P, et al (2002) Bone marrow-derived stem cells target retinal astrocytes and can promote or inhibit retinal angiogenesis. Nat Med 8(9): 1004–1010. 12145646 10.1038/nm744

[4] Espinosa-Heidmann DG, Caicedo A, Hernandez EP, Csaky KG, Cousins SW (2003) Bone marrow-derived progenitor cells contribute to experimental choroidal neovascularization. Invest Ophthalmol Vis Sci 44: 4914–4919. 14578417 10.1167/iovs.03-0371

[5] Thill M, Strunnikova NV, Berna MJ, Gordiyenko N, Schmid K, et al (2008) Late outgrowth endothelial progenitor cells in patients with age-related macular degeneration. Invest Opthalmol Vis Sci 49: 2696–2708. 10.1167/iovs.07-0955 18515596

[6] Machalinska A, Safranow K, Dziedziejko V, Mozolewska-Piotrowska K, Paczkowska E, et al (2011) Different populations of circulating endothelial cells in patients with age-related macular degeneration: a novel insight into pathogenesis. Invest Ophthalmol Vis Sci 52: 93–100. 20720219 10.1167/iovs.10-5756

[7] Riethdorf S, Fritsche H, Müller V, Rau T, Schindlbeck C, et al (2007) Detection of circulating tumor cells in peripheral blood of patients with metastatic breast cancer: A validation study of the CellSearch system. Clin Cancer Res 13: 920–928. 17289886 10.1158/1078-0432.CCR-06-1695

[8] Scholtens TM, Schreuder F, Ligthart ST, Swennenhuis JF, Greve J, et al (2012) Automated identification of circulating tumor cells by image cytometry. Cytometry Part A 81A: 138–148. 10.1002/cyto.a.22002 22170812

[9] Kraan J, Sleijfer S, Strijbos MH, Ignatiadis M, Peeters D, et al (2011) External quality assurance of circulating tumor cell enumeration using the CellSearch system: A feasibility study. Cytometry Part B 80B: 112–118. 10.1002/cyto.b.20573 21337491

[10] Shaffer DR, Leversha MA, Danila DC, Lin O, Gonzalez-Espinoza R, et al (2007) Circulating tumor cell analysis in patients with progressive castration-resistant prostate cancer. Clin Cancer Res 13(7): 2023–2029. 17404082 10.1158/1078-0432.CCR-06-2701

[11] Strijbos MH, Landburg PP, Nur E, Teerlink T, Leebeek FWG, et al (2009) Circulating endothelial cells: A potential of organ damage in sickle cell anemia? Blood Cells Mol Dis 43: 63–67. 19356955 10.1016/j.bcmd.2009.02.007

[12] Damani S, Bacconi A, Libiger O, Chourasia AH, Serry R, et al (2012) Characterization of circulating endothelial cells in acute myocardial infarction. Sci Transl Med 4(126): 126ra33. 10.1126/scitranslmed.3003451 PMC3589570 22440735

[13] Age-Related Eye Disease Study Research Group (2001) A randomized, placebo-controlled, clinical trial of high-dose supplementation with vitamins C and E, beta carotene, and zinc for age-related macular degeneration and vision loss. AREDS report no. 8. Arch Ophthalmol 119: 1417–1436. 11594942 10.1001/archopht.119.10.1417 PMC1462955

[14] Kaur H, Hainsworth DP, Caldwell CW, Hamm CW, Madsen RW (2002) Interpretation of flow cytometric measurement of lymphocytes after fluorescein angiography. Retina 22: 44–47. 11884877 10.1097/00006982-200202000-00008

[15] Seddon JM, Reynolds R, Maller J, Fagerness JA, Daly NJ, et al (2009) Prediction model for prevalence and incidence of advanced age-related macular degeneration based on genetic, demographic, and environmental variables. Invest Ophthalmol Vis Sci 50: 2044–2053. 19117936 10.1167/iovs.08-3064 PMC3772781

[16] Seddon JM, Reynolds R, Yu Y, Daly MJ, Rosner B (2011) Risk models for progression to advanced age-related macular degeneration using demographic, environmental, genetic, and ocular factors. Ophthalmology 118: 2203–2211. 21959373 10.1016/j.ophtha.2011.04.029 PMC4097877

[17] Van Craenenbroeck EMF, Conraads VMA, Van Bockstaele DR, Haine SE, Vermeulen K (2008) Quantification of circulating endothelial progenitor cells: A methodological comparison of six flow cytometric approaches. J Immunol Methods 332(1–2): 31–40. 18255093 10.1016/j.jim.2007.12.006

